# Study Design Update of the Off-pump *versus* On-pump Coronary Artery Bypass Grafting in Frail Patients: FRAGILE Trial

**DOI:** 10.21470/1678-9741-2025-0199

**Published:** 2025-07-25

**Authors:** Omar Asdrubal Vilca Mejia, Fabiane Letícia de Freitas, Bianca Meneghini, Maurilio Onofre Deininger, Rodrigo Segalote, Felipe Consentino, Mauricio Guerrieri, Alexandre Ciappina Hueb, Fernando Ribas, Rodrigo Moreira Castro, Luís Roberto Palma Dallan, Pedro GM de Barros e Silva, Renato D. Lopes, Luiz Augusto Ferreira Lisboa, John Puskas, Fabio B Jatene

**Affiliations:** 1 Instituto do Coração do Hospital das Clínicas da Faculdade de Medicina da Universidade de São Paulo (InCor-HCFMUSP), São Paulo, São Paulo, Brazil; 2 Hospital Alberto Urquiza Wanderley, Unimed, João Pessoa, Paraíba, Brazil; 3 Instituto Nacional de Cardiologia (INC), Rio de Janeiro, Rio de Janeiro, Brazil; 4 Hospital das Clínicas Samuel Libânio, Pouso Alegre, Minas Gerais, Brazil; 5 Hospital Beneficência Portuguesa de São Paulo, São Paulo, São Paulo, Brazil; 6 Brazilian Clinical Research Institute, São Paulo, São Paulo, Brazil; 7 Duke University Medical Center, Durham, North Carolina, United States of America; 8 Emory University Hospital Midtown, Atlanta, Georgia, United States of America

**Keywords:** Coronary Artery Bypass Grafts, CABG, Frailty Syndrome, Outcomes.Coronary Artery Disease

## Abstract

The Off-pump *versus* On-pump Coronary Artery Bypass Grafting in Frail Patients (FRAGILE Trial) is a multicenter, randomized controlled trial comparing off-pump and on-pump coronary artery bypass grafting in frail or pre-frail patients undergoing coronary artery bypass grafting. This manuscript presents an update to the FRAGILE Trial study design, detailing protocol modifications made in response to the time gap between the study’s conception and its actual implementation. These changes were implemented early in the trial and were formally approved by the Ethics Committee, ensuring the scientific and ethical integrity of the study and reinforcing its relevance to address a gap in a vulnerable patient population.

## INTRODUCTION

**Table:** 

Abbreviations, Acronyms & Symbols	
ARDS	= Acute respiratory distress syndrome
BCRI	= Brazilian Clinical Research Institute
CABG	= Coronary artery bypass grafting
CAM	= Confusion Assessment Method
CAM-ICU	= Confusion Assessment Method for the Intensive Care Unit
CT	= Computed Tomography
cTn	= Cardiac troponin
FRAGILE Trial	= Off-pump *versus* On-pump Coronary Artery Bypass Grafting in Frail Patients
ICU	= Intensive care unit
MI	= Myocardial infarction
ONCAB	= On-pump coronary artery bypass grafting
OPCAB	= Off-pump coronary artery bypass grafting
URL	= Upper reference limit

Fried and colleagues established frailty as an independent predictor of adverse outcomes, including falls, mobility decline, disability, hospitalizations, and mortality over a three-year follow-up period. These findings highlight frailty as a critical factor in assessing surgical risk, particularly in the context of cardiac surgery^[1^,^2]^.

Despite advances in surgical techniques and perioperative care, the debate surrounding the superiority of off-pump coronary artery bypass grafting (OPCAB) *vs.* on-pump coronary artery bypass grafting (ONCAB) persists, particularly for pre-frail and frail patients. Existing randomized trials, such as CORONARY^[3]^, ROOBY^[4]^, GOPCABE^[5]^, and the Beating Heart versus Conventional Coronary Artery Bypass Surgery (or BBS)^[6]^, have primarily focused on low-, intermediate-, and high-risk patients. However, patients with frailty criteria have been notably underrepresented in these studies, leaving a significant gap in high-quality evidence for this population. While a recent study attempted to address outcomes in frail patients, the lack of randomization limits the strength of its conclusions^[7]^.

To address this gap, we proposed the Off-pump *versus* On-pump Coronary Artery Bypass Grafting in Frail Patients (FRAGILE Trial), to compare clinical outcomes in a randomized fashion of OPCAB *vs.* ONCAB, specifically in frail patients. This trial aims to generate robust evidence to guide clinical decision-making and optimize surgical outcomes for this vulnerable and growing population. However, this study was originally designed and published in 2017 and underwent modifications in its design at the time of implementation (after the inclusion of the first 10 patients), in 2019. Therefore, the present manuscript aims to highlight the previously published protocol changes and to ensure that these modifications will not affect future publication, as they were made at the very beginning of the study and were carried out with the approval of the local Ethics Committee.

## METHODS

### Study Design Update

The FRAGILE Trial (ClinicalTrials.gov: NCT02338947)^[8]^ is a randomized, controlled, and multicenter study that recruited frail patients who underwent coronary artery bypass grafting (CABG). Eligible, consenting patients were enrolled and randomized to ONCAB or OPCAB. The study was conducted at six hospitals in Brazil, all with extensive experience in ONCAB and OPCAB surgeries. The enrollment was from January 2019 to December 2024 and included patients classified as pre-frail and frail.

### Primary Endpoint

The new primary endpoint was defined as a composite of mortality rates (all-cause death) and perioperative complications, including acute myocardial infarction, stroke, renal failure, acute respiratory distress syndrome (ARDS), and bleeding reoperation, occurring within 30 days after surgery. The scientific committee understood that the previous primary objective, which included variables as cardiac arrest with successful resuscitation, low cardiac output syndrome/cardiogenic shock and coronary reintervention, would not include metrics that could be more related to the use of extracorporeal circulation in frail patients. For this reason, the variables renal failure, ARDS, and reoperation due to bleeding were included. This would better reflect the systemic impact of extracorporeal circulation in frail patients, justifying our hypothesis. The components and definitions of the primary endpoint are presented in Table 1.

**Table 1:** Primary endpoint components and definitions.

Mortality and Perioperative Complications
The primary outcome comprises:
• All-cause death: from the date of surgery until 30 days postoperatively, assessed over a period of up to 30 days.
• Major perioperative complications: major complications occurring from the date of surgery until 30 days postoperatively, assessed over a period of up to 30 days.
Definitions of Major Complications
1. Myocardial infarction (MI)
• The latest Universal Definition of Myocardial Infarction was considered, being type 5 MI within 48 hours after surgery and type 1 MI between 48 hours and 30 days after surgery.
• Criteria for coronary artery bypass grafting (CABG)-related MI ≤ 48 hours after the index procedure (type 5 MI): CABG-related MI is arbitrarily defined as elevation of cardiac troponin (cTn) values > 10 times the 99th percentile upper reference limit (URL) in patients with normal baseline cTn values. In patients with elevated preprocedure cTn in whom cTn levels are stable (≤ 20% variation) or falling, the postprocedure cTn must rise by > 20%. However, the absolute postprocedural value still must be > 10 times the 99th percentile URL. In addition, one of the following elements is required:
• Development of new pathological Q waves^*^;
• Angiographic documented new graft occlusion or new native coronary artery occlusion;
• Imaging evidence of new loss of viable myocardium or new regional wall motion abnormality in a pattern consistent with an ischemic etiology.
2. Neurological complications
• Stroke: defined as a neurological deficit lasting > 24 hours with positive findings on computed tomography (CT).
• Transient ischemic attack: defined as a neurological deficit lasting < 24 hours with positive findings on CT.
3. Renal insufficiency
Defined as:
• An increase in plasma creatinine > 2 mg/dL associated with
• A urinary output < 0.5 mL/kg/h over 12 hours.
4. Acute respiratory distress syndrome
Defined as the presence of:
• Tachypnea (respiratory rate > 30 breaths/min);
• Bilateral pulmonary infiltrates on chest radiography;
• Severe hypoxemia (arterial oxygen partial pressure/inspired oxygen fraction < 200);
• The need for positive end-expiratory pressure > 5 cmH₂O;
• No evidence of left ventricular failure (pulmonary capillary wedge pressure < 18 mmHg); and
• No other pathological findings to explain these observations.
5. Reoperation for bleeding
Defined as the need for chest reopening due to bleeding, as evidenced by:
• 500 mL of blood drainage from the chest tube within the first hour;
• 400 mL within the second hour;
• 300 mL within the third hour, or
• Total bleeding > 1000 mL within the fourth hour.

* Isolated development of new pathological Q waves meets the type 5 MI criteria if cTn values are elevated and rising but < 10 times the 99^th^ percentile^[17]^

All the primary outcomes will be adjudicated by the Brazilian Clinical Research Institute (BCRI), an independent clinical events committee, blinded to the randomization arm.

### Secondary Outcomes

The secondary endpoints included operative time, mechanical ventilation time, hyperdynamic shock, new onset of atrial fibrillation, need for pacing > 24 hours, renal replacement therapy, pneumonia, length of stay in the intensive care unit, length of stay in the hospital, transfusion, recurrence of angina, rate of complete revascularization, and evolution of frailty status, all assessed within 30 days after surgery, except for frailty status, which was evaluated at baseline, six months, one and two-year post-discharge (Table 2 and Table 3).

**Table 2:** Secondary endpoint components and definitions.

Secondary Outcome Measures
1. Operative time
Defined as the total time from the start of anesthesia induction to the end of the surgical procedure, measured in minutes.
2. Mechanical ventilation time
Defined as the total time of mechanical ventilation, measured in hours, from initiation in the operating room or intensive care unit (ICU) to the time of successful extubation.
3. Hyperdynamic shock
Defined as a cluster of symptoms signaling the onset of septic shock, often including shaking chills, rapid rise in temperature, flushing of the skin, galloping pulse, and alternating rise and fall of blood pressure. Assessed within 30 days after surgery.
4. New onset of atrial fibrillation
Diagnosed via 12-lead electrocardiography to confirm the occurrence of new onset atrial fibrillation within 30 days after surgery.
5. Need for pacing > 24 hours
Defined as patients requiring pacing for > 24 hours within 30 days after surgery.
6. Renal replacement therapy
Defined as any type of renal replacement therapy initiated in a patient who was not previously dependent on it, assessed within 30 days after surgery.
7. Pneumonia
Diagnosed by a physician or advanced practitioner based on laboratory findings (*e.g.*, positive sputum culture from transtracheal fluid or bronchial washings) and/or radiological evidence (*e.g.*, chest radiography showing pulmonary infiltrates), assessed within 30 days after surgery.
8. Length of stay in ICU
Defined as the total time spent in the ICU, measured in hours, assessed within 30 days after surgery.
9. Length of stay in hospital
Defined as the total time spent in the hospital, measured in days, assessed within 30 days after surgery.
10. Transfusion requirement
Defined as the number of units of blood transfused, assessed within 30 days after surgery.
11. Recurrence of angina
Assessed based on the Canadian Cardiovascular Society classification for angina recurrence within 30 days after surgery.
12. Rate of complete revascularization
Defined as the proportion of patients achieving complete revascularization, evaluated at the end of the surgical procedure.
13. Evolution of frailty status
Frailty status will be assessed based on the Fried Frailty Criteria, which include five components: unintentional weight loss, exhaustion, weakness (grip strength), slowness (gait speed), and low physical activity. Patients will be categorized as robust (0 criteria), pre-frail (1-2 criteria), or frail (3-5 criteria). Changes in frailty status will be evaluated at baseline and at follow-up to determine progression, stability, or improvement, assessed at six months and one year.

**Table 3:** Other pre-specified outcomes components and definitions.

1. Delirium
Defined as delirium assessed using the Confusion Assessment Method for the Intensive Care Unit (CAM-ICU) in the ICU and the Confusion Assessment Method (CAM) in the ward. According to the CAM-ICU instrument, delirium is diagnosed when features 1 and 2 are positive, along with either feature 3 or 4. The diagnosis of delirium requires the presence of baseline mental status changes, inattention, altered level of consciousness, or disorganized thinking. Patients will be evaluated with the CAM-ICU during their stay in the ICU and with the CAM after being transferred to the ward. This outcome will be evaluated throughout the duration of the hospital stay.
2. Neurobehavioral outcomes after cardiac surgery
Defined as the global cognitive status evaluated through a test consisting of 30 simple questions and tasks covering several domains, including orientation in time and place, repeating and recalling lists of words, arithmetic, language use and comprehension, and non-verbal memory. This outcome will be assessed at six months and one year following the surgical procedure.
3. Quality of life after cardiac surgery
Defined as the quality of life assessed using the EuroQol registration, which includes five domains and a visual analogue scale. The best imaginable state is marked by 100, and the worst imaginable state is marked by 0. This outcome will be evaluated at six months and one year postoperatively.
4. Cost
Defined as the adjusted amount in US dollars of the total cost of coronary artery bypass surgery, evaluated only at the coordinating center (Instituto do Coração/Hospital das Clínicas da Faculdade de Medicina da Universidade de São Paulo). This outcome will be analyzed at one year following surgery.
5. Graft patency
Defined as the patency of grafts and coronary artery disease at one year of follow-up, evaluated by coronary computed tomography angiography. This outcome will be assessed at one year postoperatively.
6. Clinical and angiographic scores correlation with prognostic
Defined as the clinical correlation between the revascularization strategy and the usefulness of the following scores for prognostic evaluation: Synergy Between Percutaneous Coronary Intervention With TAXus and Cardiac Surgery Score (or SYNTAX score); Age, Creatinine, and Ejection Fraction Score (or ACEF score); Clinical SYNTAX score; European System for Cardiac Operative Risk Evaluation II (or EuroSCORE II); The Society of Thoracic Surgeons risk model; and the Prediction Index of the Instituto do Coração (or InsCor). This outcome will be evaluated at one year postoperatively.

### Eligibility

#### Inclusion Criteria

The inclusion age criterion was revised from 65 to 60 years, based on the observation of a high prevalence of frailty beginning at this earlier age range. Patients classified as pre-frail or frail according to the Fried Frailty Criteria (scores 1-2 and 3-5, respectively), with an indication for CABG and eligible for surgery using either technique (ONCAB or OPCAB) were included, after the Heart Team definition.

#### Exclusion Criteria

The exclusion criteria included: patients with an indication for an additional procedure (associated surgery) besides myocardial revascularization; those requiring emergency surgery, defined as the need for surgical intervention within the first 24 hours of hospitalization; patients with a history of previous cardiac surgery, regardless of the surgical approach, including non-median sternotomy; individuals with a creatinine clearance < 30 mL/min, calculated using the Cockcroft & Gault formula; patients with glycated hemoglobin (or HbA1c) levels > 8.5%; the presence of a porcelain aorta, primarily identified by computed tomography or chest X-ray; and patients who did not provide written informed consent to participate in the study.

### Fried Frailty Phenotype

Patients underwent screening based on the five criteria established by Fried, known as the Frailty Phenotype^[9]^.

### Randomization, Allocation Concealment, and Blinding

Patients were randomized 1:1 to OPCAB or ONCAB using Research Electronic Data Capture (or REDCap)^[10]^, in blocks of 10, stratified by center. Patients, outcome assessors, and statisticians were blinded; surgeons, anesthesiologists, and intensivists remained unblinded to technique but adhered to standardized protocols.

### Surgical Procedures: Off-pump and On-pump Coronary Artery Bypass Grafting Protocols

Both OPCAB and ONCAB procedures followed standardized techniques as published previously to ensure consistency across centers. All centers had surgeons with > 100 OPCAB cases of experience.

### Follow-up

Evaluations occurred preoperatively and at 30 days (remote/in-person), six and 12 months (in-person), and 24 months (remote) post-discharge. Preoperative assessment included frailty phenotype, Mini-Mental State Examination (or MMSE), EuroQol Five Dimensions-Five Levels, depression screening, and Charlson Comorbidity Index. Postoperative visits assessed outcomes, frailty status, cognitive function, quality of life, and rehospitalization. The evaluation of graft patency following CABG was initially planned to be conducted at six months postoperatively. However, after a more comprehensive review of the current literature, this timepoint was revised to 12 months. The majority of scientific articles and clinical studies in this field assess graft patency at the one-year mark, which is considered a more standardized and clinically relevant period for evaluating mid-term outcomes. Therefore, in order to align our methodology with established research practices and enhance the comparability of our results, the evaluation period was adjusted accordingly^[11]^.

### Statistical Analysis

Initially, the sample size was calculated to include 630 patients^[12]^. However, following a modification of the study's primary outcome, it became necessary to recalculate the required sample size to align with the revised objectives. The change in the primary objective occurred after the inclusion of the first 10 patients. It is worth noting that the primary endpoint chosen for the FRAGILE Trial was coincidentally the same as the one used in the previous study^[13]^. The sample size was determined using the G*Power software (version 3.1.9.7)^[14]^. To ensure statistical rigor and to account for potential participant attrition during the study, the recalculation maintained an allocation ratio of 1:1. Considering a primary outcome rate of 13.3% in high-risk patients undergoing ONCAB and 5.8% in those undergoing OPCAB^[15]^, with a statistical power of 85% and a significance level of 5%, the revised calculation indicated a requirement of 220 patients per group, resulting in 440 participants for the study.

## CONCLUSION

In summary, the adjustments made to the FRAGILE Trial protocol were implemented promptly, after the first 10 patients were enrolled, and fully endorsed by the local Ethics Committee, ensuring that they did not alter the core study or compromise the methodological integrity of the trial. Consequently, these early modifications have no bearing on the validity of the main study findings. By preserving the original randomization framework and outcome assessments, the FRAGILE Trial remains well-positioned to generate robust, clinically meaningful data comparing OPCAB and ONCAB specifically in frail and pre-frail patients. The forthcoming results will therefore fill a critical evidence gap, guiding surgeons and care teams toward optimized revascularization strategies for this specific population.
